# Face inversion effect and exposure duration on age classification accuracy

**DOI:** 10.1177/03010066251346116

**Published:** 2025-06-10

**Authors:** Janice Attard-Johnson, Jack Clifton, Alejandro J. Estudillo

**Affiliations:** Department of Psychology, Faculty of Science and Technology, 6657 Bournemouth University, Fern Barrow, Poole, UK; Department of Psychology, Faculty of Science and Technology, 6657 Bournemouth University, Fern Barrow, Poole, UK; Department of Psychology, Faculty of Science and Technology, 6657 Bournemouth University, Fern Barrow, Poole, UK

**Keywords:** Face inversion effect, age perception, exposure duration, face processing

## Abstract

The effect of face orientation and exposure duration on facial identity recognition and matching are well-documented but has scarcely been examined for facial age perception. Using a facial age categorisation task (i.e., classifying faces as over and under the age of 18) with ambient faces, we manipulated facial orientation (upright and inverted) and exposure duration (250 and 2000 ms) to examine their unique and interactive effects on age classification accuracy. Across two experiments, age classification accuracy was impaired by inverting faces. Additionally, overall accuracy was improved when participants were required to view the faces for the full length of the long duration (2000 ms) (experiment 2), but not when they had the opportunity to respond earlier than the end of stimuli (experiment 1). However, there was no interactive effect of exposure duration and facial orientation. These findings suggest that accurate age classification relies on facial information that is disrupted when inverted.

## Introduction

The Face Inversion Effect (FIE) is a well-documented effect characterised by an observable reduction in performance in accuracy on a face processing task when the faces are inverted compared to upright faces (Gerlach & Mogensen, 2024; Rakover, 2013; Yin, 1969). The mechanisms underlying the effect are debated (Gerlach et al., 2023; Gerlach & Mogensen, 2024), however researchers argue that this impairment stems, at least in part, from a disruption in the ability to use holistic processing when faces are inverted (see Rakover, 2013). Further, some studies show that additional viewing time reduces impairment caused by inversion for facial identity (e.g., Richler et al., 2011). The present study examined the effect of orientation and exposure duration on a cognitive process that has received comparatively less attention: facial age perception.

Existing theoretical models of face processing distinguish between invariant and changeable aspects of faces (Bruce & Young, 1986; Burton et al., 1999; Haxby et al., 2000). While invariant aspects of faces include features linked to identity, such as sex and race, changeable aspects of faces are relatively identity-independent and play a more significant role in social communication, such as emotional expression (Haxby et al., 2000). As age transforms facial features progressively over an individual's lifespan it provides a unique combination of both stability and changeable characteristics. However, there is uncertainty around whether age is more appropriately classified within the same category as invariant (i.e., sex and race) or variant face-aspects (i.e., emotional expression) (Haxby et al., 2000). There is currently insufficient theoretical and empirical evidence on the cognitive architecture of facial age perception to draw any conclusions about its relationship to other face processing tasks. Consequently, it is unclear whether the same phenomenon observed for facial identity processing and other more widely researched non-identity-specific characteristics will also extend to facial age perception. One robust effect in the face processing literature is the Face Inversion Effect (FIE), and examining whether this effect is also present for facial age perception may be able to provide some insights on the nature of the relationship between age perception and other face processing mechanisms.

The Face Inversion paradigm contributes to two key theoretical debates. First is a comparison of the inversion effect in faces and objects, such that the inversion effect is found to disproportionately affect face compared to object processing (Rakover, 2013; Yin, 1969). This finding is taken as evidence for specialised face processing cognitive mechanisms in humans (Burke & Sulikowski, 2013; Rakover, 2013), although recent studies challenge this view (Gerlach et al., 2023). Second is a comparison of upright and inverted faces for face-specific cognitive processes, such as expression recognition (Bimler et al., 2013), gender discrimination (Bruce et al., 1993), face matching (Farah et al., 1995), and face recognition (Kanwisher et al., 1998; Leder & Bruce, 2000; Yovel & Kanwisher, 2005), which centres around the debate between the global/configural and featural processing of faces. Inversion strongly disrupts the discrimination of faces differing on configural information (i.e., spatial distance between the facial features), but the FIE effect is smaller for faces differing only on specific facial features and is absent for faces differing in the colour of facial features (Leder & Carbon, 2006). These findings suggest a prominent role of global/configural information on face identification (see also DeGutis et al., 2013; Estudillo et al., 2022; Lee et al., 2022; Rossion, 2008).

Certain ‘global’ cues are important for age judgements, such as changes to bone structure, distribution of subcutaneous fat, muscle tone, and cardioidal strain (see Rhodes, 2009; Swift et al., 2021)—all of which may affect facial configuration. However, some other age-related changes are more ‘featural’, such as skin tone, wrinkles and contrast which contribute up to 33% of the information in the assessment of age (González-Alvarez & Sos- Peña, 2023; Porcheron et al., 2013), as well as thinning of lips, and changes to cartilage in nose and ears (Sforza et al., 2011). The individual contribution of featural and global cues on human age perception is not well defined. If facial age relies more heavily on global cues, then age estimation may become disrupted with face inversion. In contrast, if featural cues are more dominant, then the processing of these cues would not be disrupted, and the FIE would be absent providing evidence for more featural-based processing of age perception.

Examination of the FIE on facial age perception could provide valuable insights into the roles of global and featural information in this process. Although a composite face study suggests that age is processed holistically (Hole & George, 2011), another study found that inversion did not affect facial age estimation accuracy suggesting that age processing leans more featural (George & Hole, 2000). Differences in methodological approaches and measurement factors have been found to impact the strength and presence of the FIE in wider face processing studies (for reviews see, Rakover, 2013), so it is conceivable that different paradigms underlie differences in FIE patterns.

Additionally, longer viewing times are found to reduce the FIE (see e.g., Barton et al., 2001; Richler et al., 2011). George and Hole's (2000) age-estimation inversion study was self-paced and the average response duration response is not reported. Increased exposure time may provide additional time for faces to eventually be processed holistically (Richler et al., 2011) or to switch strategy allowing different face information to be processed in more detail (Özbek & Bindemann, 2011), potentially masking the FIE. There is no published work examining the unique effect of exposure duration, as well as its interaction with facial orientation, on facial age assessment. Therefore, the question of whether age perception accuracy will be impaired by face inversion, and whether this effect will be attenuated with additional viewing time, remains unanswered.

To address these questions, we conducted two simple experiments examining the unique effects of orientation (upright/inverted) and exposure duration (250 ms/2000 ms), as well as any interactive effects, on the age classification of adolescent faces. In experiment 1, responses could be made as soon as participants felt that they had reached an accurate decision within the presentation window. In experiment 2, participants could only enter their decision once the presentation window had elapsed, thus requiring participants to use the *full* exposure time to consider their decision. The reason for this is to differentiate between the point at which it was felt by the participant that a decision could accurately be made, and whether it is possible to further improve accuracy by constraining response time and thus ‘forcing’ longer exposure time.

## Experiment 1

### Method

#### Participants

We recruited 47 undergraduate student participants (39 female) (M = 19.8 years, range = 18 to 23, SD = 1.17) through the University's research management system and they received course credit for their time. All but four participants selected ‘White’ for their ethnicity, one selected ‘South Asian’, one selected ‘Black or African’ and two selected ‘Other’. A sensitivity power analysis conducted with the software MorePower 6.0 (Campbell & Thompson, 2012) revealed that the present sample size was sufficient to detect a large inversion effect (p^2^ = .15). Ethical approval was obtained from the Institution's Ethics Board (Ref: 47626), and all participants provided written consent to take part.

#### Stimuli and Procedure

The Age Categorisation Task (ACT) used in Attard-Johnson et al. (2024) was adapted for this experiment. The ACT is analogous to real-world age verification scenarios requiring participants to classify faces as under or over the age of 18. Age perception tasks often require participants to enter a precise numeric estimation (e.g., Hole & George, 2011) which is not ideal for manipulating exposure duration. The binary response of the ACT overcomes this limitation and was therefore considered a suitable paradigm for this study. The 32 images were selected from an existing database (Murray et al., 2022) depicting natural photographs of Caucasian male and female faces (16 each) aged between 14 and 21 (excluding 18 years) with half between 14 and 17, and the remaining half between 19 and 21. Faces were all in colour and varied naturally but they were all front profiles and cropped around the head to exclude the neck. Images were resized to a set width of 230 pixels, though the height was allowed to vary to minimise distortion. Further, half of all the faces were presented upright and inverted, and with a presentation time of either 250 or 2000 ms split equally across gender, age category and rotation of the images. The durations were chosen to represent a mid- and long-exposure range (e.g., Barton et al., 2001; Richler et al., 2011). Four versions were created to counterbalance the experimental conditions such that each face was presented either upright/250 ms, upright/2000 ms, inverted/250 ms or inverted/2000 ms across the different versions. Participants were randomly assigned to one of the four versions and thus were only exposed to each face once.

The experiment was conducted on the online testing platform *Testable* (testable.org) which uses a robust calibration feature to adjust the experiment to the size of the participant's monitor. Participants completed the task remotely, and the task could only be accessed on a computer and could not be completed on a phone or tablet. Participants were instructed to be as accurate as possible. Each image was displayed for the prescribed duration and participants could respond at any point *during* or *after* the presentation window (either 250 or 2000 ms) had elapsed. Participants entered a response by pressing ‘u’ if they thought the face was under the age of 18, or ‘o’ if the face was over the age of 18. An inter-trial fixation cross was presented in the centre of the screen for 800 ms. There were a total of 32 trials which were presented in a randomly generated order for each participant.

### Results and Discussion

For this analysis, we conducted a traditional repeated measures analysis of variance (ANOVA) and the equivalent Bayesian ANOVA in JASP (version 0.18.3). We report Bayes Factor 10 (*BF*_10_) for main effects which represents how likely it is for the data to arise under the alternative model compared to the null model. For the interaction term, we report the effects for the Inclusion Bayes Factor (BF_inc_) across matched-models which reflects the evidence for the interaction model stripped of other effects (see Mathôt, 2017). A Bayes Factors of greater than 3 represents substantial support for the alternative hypothesis and smaller than 0.3 represents substantial support for the null hypothesis, and a BF in-between 0.3 and 3 represents only weak or anecdotal evidence (Jeffreys 1961; Wetzels et al., 2011).

#### Accuracy

**Figure 1.**
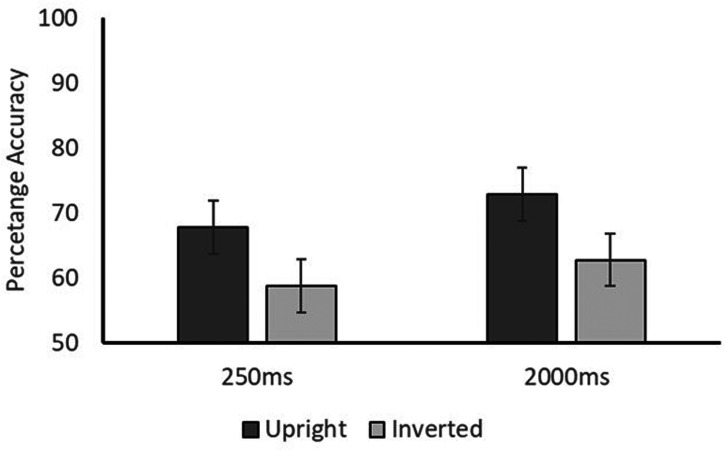
Illustration of percentage accuracy for experiment 1. Vertical lines show 95% confidence intervals.

Figure 1 illustrates the percentage accuracy across each condition. A 2 (Orientation: Upright, Inverted) × 2 (Maximum exposure duration: 250 ms, 2000 ms) repeated measures ANOVA revealed a main effect of orientation, *F*(1,46) = 23.966*, p* < .001, p^2^ = 0.343 (BF_10_ = 246.201), recording a 9.5% reduction in accuracy for the classification of inverted faces compared to upright faces. No main effect of maximum exposure duration, *F*(1,46) = 3.166*, p* = .082, p^2^ = 0.064 (BF_10_ = 0.725), or an interaction, *F*(1,46) = 0.053*, p* = .818, p^2^ = 0.001 (BF_inc_ = 0.233), was found.

#### Response Times

Although RTs were not the focus, we analysed RTs for completeness and to explore differences in decision time. Four outliers (greater than mean +/− 3*standard deviation) were replaced with the next maximum value to reduce any undue influence. A 2 (Orientation: Upright, Inverted) × 2 (Maximum exposure duration: 250 ms, 2000 ms) repeated measures ANOVA revealed a main effect of exposure duration, *F*(1,46) = 10.663*, p* = .002, p^2^ = 0.188 (BF_10_ = 14.312), whereby responses for the 250 ms presentation condition were faster (M = 1230 ms) compared to the 2000 ms presentation condition (M = 1521 ms). No other main effects, *F*(1,46) = 0.065*, p* = .799, p^2^ = 0.001 (BF_10_ = 0.196), or interactions, *F*(1,46) = 2.889, *p* = .096, p^2^ = 0.059 (BF_inc_ = 1.023), were found.

Thus, face inversion negatively impacted face classification accuracy and additional time did not lessen this effect. However, participants were given *up to* the maximum exposure duration of 2000 ms to respond, and RT analysis revealed that participants generally did not utilise the full duration. Experiment 2 will examine whether restricting participants to view the image for the full duration, by withholding the response window until after the stimulus has been removed, improves accuracy.

## Experiment 2

### Method

#### Participants

Using a similar sample size and sampling method to experiment 1, and a total of 49 participants (41 female, 2 ‘other’) were recruited (M = 21.51 years, range = 18 to 52, SD = 6.89). All but six participants selected ‘White’ for their ethnicity, two selected ‘South Asian’, one selected ‘Hispanic or Latino’ and three selected ‘Other’. Participants were an independent group to those who partook in experiment 1.

#### Stimuli and Procedure

The task was identical to experiment 1, but with one key difference. Participants could only enter their response *after* the images were removed from view thus restricting participants to view the images for the full duration.

#### Results

**Figure 2.**
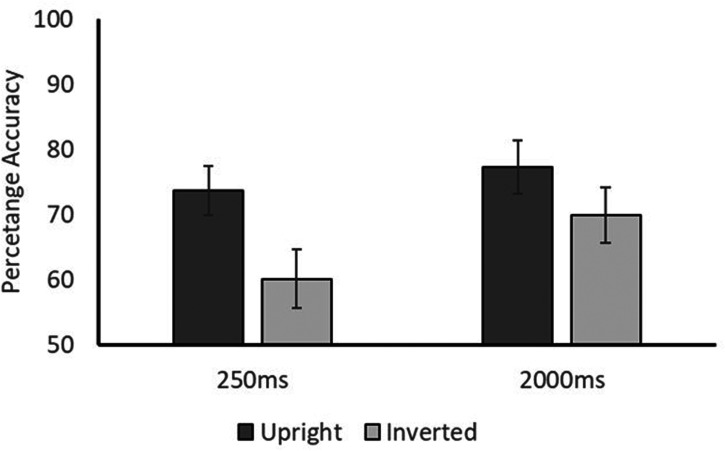
Illustration of percentage accuracy for experiment 2. Vertical lines show 95% confidence intervals.

Figure 2 illustrates the percentage accuracy across each condition. A 2 (Orientation: Upright, Inverted)×2 (Exposure duration: 250 ms, 2000 ms) repeated measures ANOVA revealed a main effect of orientation, *F*(1,48) = 22.046*, p* < .001, p^2^ = 0.315 (BF_10_ = 724.708), such that there was a 10.5% reduction in accuracy for the classification of inverted faces when compared to upright faces. There was a main effect of exposure duration, *F*(1,48) = 9.171*, p* = .004, p^2^ = 0.160 (BF_10_ = 7.779), such that there was a 9.6% increase in accuracy for the longer duration. The analysis did not find a significant interaction effect, *F*(1,48) = 2.658*, p* = .110, p^2^ = 0.052 (BF_inc_ = 0.870).

## Discussion

The effect of face orientation and exposure duration on facial age perception has rarely been examined. Here we manipulated orientation (inverted/upright) and exposure duration (250 ms/2000 ms) for a dichotomous ACT which required participants to categorise adolescent and young adult faces as over or under the age of 18. Overall, performance accuracy for upright faces was approximately 73%, and similar to the previous study using the ACT (78%; Attard-Johnson et al., 2024). Across both experiments, inverted faces recorded an average 10% reduction in performance accuracy, indicating the presence of the FIE for facial age perception. Contrary to our prediction, exposure duration did not interact with the FIE to attenuate it. However, there was an overall improvement in performance accuracy when participants were *required* to view the face for the full longer duration before entering their decision (experiment 2), but not when they were given the *opportunity* to view the face for longer with the option to respond earlier (experiment 1).

Both experiments found an effect of face orientation which suggests that the FIE is present for facial age estimation using an age classification task comprising adolescent and young adult faces. According to the theoretical framework of FIE, this suggests that accurate age perception, at least for these age groups, relies on facial information that is disrupted when inverted. If we assume, as is generally accepted (e.g., Leder & Carbon, 2006; Rossion, 2008) that face inversion impairs global/configural processing of faces, our results suggest that age perception primarily relies on this type of processing. Interestingly, as shown in Figures 1 and 2, performance in the inverted condition remained above chance, suggesting that reliance on individual facial features can still provide sufficient information to support age perception.

These findings are in contrast with George and Hole's (2000) experiment which did not find an FIE for numerical estimation of facial age. This suggests that the way we measure facial age matters and that different tasks (e.g., numeric estimation vs classification) might probe different cognitive processing mechanisms (see Attard-Johnson et al., 2024) or that different tasks rely on different use of facial information of which some may be more impacted by inversion (see e.g., Kuraguchi & Nittono, 2023). An alternative explanation might be related to the age of the stimuli. In this study we used young adult and adolescent faces in a real-world simulated task. Facial cues used in age perception for younger faces might be different to those relied upon for estimating the age of older faces. Specifically, changes to distribution of fat and cardioidal strain, the more ‘global’ cues are features which are particularly related to growth (up to the age of 20) (Rhodes, 2009; Swift et al., 2021) and less so in adulthood when more ‘featural’ cues, such as wrinkles, skin tone, thinning of lips are more pronounced (González-Alvarez & Sos-Peña, 2023; Porcheron et al., 2013; Sforza et al., 2011). Younger faces may therefore be more susceptible to the FIE, and these findings may not generalise to older age groups. Future work should consider systematically comparing the FIE across younger and older faces.

Experiment 1, which allowed participants to respond before the full exposure window closed, did not find an effect of exposure duration. However, experiment 2 required participants to view the faces for the full durations, and here we recorded an increase in performance accuracy for both upright and inverted faces. Positive effects of longer exposure durations on facial identity recognition are well documented (see for example, Memon et al., 2003). This suggests that additional time may prompt participants to adopt a different processing strategy, possibly studying the faces in more detail (Özbek & Bindemann, 2011), however additional viewing time did not interact with face orientation specifically to attenuate the FIE in this study.

In conclusion, accurate classification of facial age of adolescent and young adult faces was impaired by face inversion. Additionally, a longer viewing time of 2000 ms (compared to 250 ms) improved overall age classification accuracy for both upright and inverted faces. These findings suggest that the processing of adolescent and young adult faces rely, at least to some degree, on features which are disrupted by face inversion. This study offers a first investigation into the FIE for facial age perception, and further research is needed to determine whether these findings extend to older faces and whether even longer exposure durations can have additional positive effects on the FIE.
